# Contezolid, a novel oxazolidinone antibiotic, may improve drug-related thrombocytopenia in clinical antibacterial treatment

**DOI:** 10.3389/fphar.2023.1157437

**Published:** 2023-04-24

**Authors:** Bi Li, Ying Liu, Jiaqi Luo, Yun Cai, Mengli Chen, Tianlin Wang

**Affiliations:** ^1^ Department of Pharmacy, Medical Supply Center of Chinese PLA General Hospital, Beijing, China; ^2^ Medical School of Chinese PLA General Hospital, Beijing, China

**Keywords:** new oxazolidinone antibiotics, contezolid, drug-induced thrombocytopenia, efficacy and safety, case series

## Abstract

One of the major limitations in the clinical use of existing oxazolidinone antibiotics is their characteristic adverse reactions, in particular thrombocytopenia. In anti-infective treatment, if patients are suspected of having drug-induced thrombocytopenia, the first step is to immediately discontinue the offending drug. Even in patients with severe infections, the antibacterial drug may need to be changed or the antibacterial treatment may need to be discontinued because thrombocytopenia may have a more serious clinical prognosis. In addition, if the patient needs to continue antibacterial treatment after discharge, the lack of conditions for monitoring platelet levels may also pose hidden dangers to the patient. Contezolid is an orally administered oxazolidinone antibacterial agent approved by the National Medical Products Administration of China in 2021. We found that contezolid may have an improved safety profile with a significantly reduced potential for myelosuppression based on the results of our observational clinical study. In this article, we review the advantages of contezolid as a new oxazolidinone antibiotic and describe three typical clinical cases of patients who experienced drug-induced thrombocytopenia after using linezolid. The platelet levels of these different patients were all significantly improved to varying degrees after initiation of contezolid treatment.

## 1 Introduction

Oxazolidinones are a class of synthetic antibiotics with a chemical structure characterized by a basic 2-oxazolidinone nucleus. As oxazolidinones bind to the 50 S ribosomal subunit, thereby inhibiting the biosynthesis of bacterial proteins, they are active against a broad spectrum of multidrug-resistant Gram-positive bacteria (GPB), including vancomycin-resistant *Enterococcus* (VRE), methicillin-resistant *Staphylococcus aureus* (MRSA), and *Mycobacterium tuberculosis* (Diekema and Jones, 2001). Although this is a classic type of antibiotic compound that has been used for many years, only two of these oxazolidinone antibiotics are clinically approved at present. The first oxazolidinone antibacterial drug, linezolid, was approved by the U.S. Food and Drug Administration (FDA) in 2000 (Wilcox, 2005) for the treatment of infectious diseases, such as skin and subcutaneous tissue infections, pneumonia, VRE infections, and other infectious diseases caused by multiple GPB. The second-generation oxazolidinone, tedizolid, was approved for clinical use in 2014. The development of new oxazolidinone antibiotic structures has continued, with radezolid and sutezolid currently in research and development.

One of the major limitations in the clinical use of oxazolidinone antibiotics is their characteristic adverse reactions, including thrombocytopenia, leukopenia, pancytopenia, anemia, and serotonin toxicity. These reactions are due to their effects on myelosuppressive and monoamine oxidase (MAO) inhibitory effects (Slatter et al., 2001). Of note, oxazolidinones inhibit the initiation of protein synthesis by preventing the formation of the tRNAfMet-mRNA-70 S (or 30 S) subunit ternary complex (Swaney et al., 1998) due to the homology between the 23 S RNA target in prokaryotes and the mitochondrial protein synthesis machinery in mammals (Vinh and Rubinstein, 2009). Consequently, due to adverse reactions such as thrombocytopenia, discontinuation of its application is common (Singh et al., 2019). Taken together, the antibacterial effect and adverse reactions of oxazolidinone antibiotics almost coexist. When comparing the risk of thrombocytopenia with linezolid and tedizolid in real-world comparative safety studies using FDA Adverse Events Reporting System data, no significant differences were observed between these two antibiotics (Lee and Caffrey, 2017). The vicious cycle of “drug-induced thrombocytopenia—change in drug regimen—prolonged antibacterial course” and the almost unavoidable myelosuppressive toxicity caused by existing oxazolidinone drugs make the development of new drug-resistant antibacterial agents particularly urgent. Consequently, the development of new oxazolidinone antibiotic structures has continued (Barbachyn and Ford, 2003).

Contezolid, a new oxazolidinone antibacterial agent, was approved by the National Medical Products Administration of China in June 2021 (Hoy, 2021) for the treatment of complicated skin and soft tissue infections, including, but not limited to, infections involving MRSA, *Streptococcus pyogenes,* and *Streptococcus agalactiae*. Here, we review the treatment benefits of contezolid and report on its good antibacterial efficacy and improved platelet counts in patients clinically treated with contezolid. In particular, we review the structure–activity relationship of oxazolidinone antibiotics and the structural characteristics of contezolid, the current *in vitro* research on the antibacterial activity of contezolid, and research on the myelosuppression caused by oxazolidinones compared to the safety of contezolid. We also present a case series demonstrating the efficacy and safety of contezolid in clinical use.

## 2 Chemical structure characteristics

**FIGURE 1:**
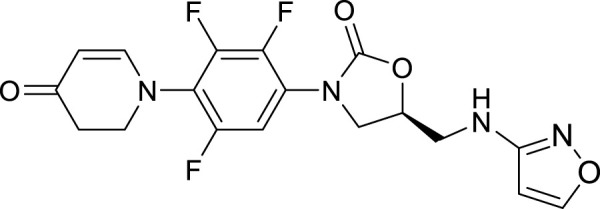
Chemical structure of contezolid.

Structure–activity relationship studies of existing oxazolidinone antibiotics have shown that both the 5-R configuration on the A ring and the B ring of the N-aryl group of oxazolidinone compounds are both necessary for antibacterial activity (Zhao et al., 2021). This combination of structures, interacting with regions of the peptidyl transferase center (PTC) binding site, is related to the potency of these antibiotics against all bacterial strains (Nagiec et al., 2005). In the structure of linezolid, the A and B rings are in the same plane (Eyal et al., 2015). In contrast, as shown in Figure 1, in contezolid, the A and B rings have a non-coplanar structure, which is obtained by three fluorine atom substituents in the B ring’s amine structure (Gordeev and Yuan, 2014). The isoxazole-substituted keto group also appears to have provided structural advantages to contezolid. The isoxazole in the C5-domain of contezolid improves its chimerism into a hydrophobic pocket defined by the PTC of the 50 S ribosomal subunits A2451 and C2452, which may increase the binding of contezolid to ribosomes (Wright et al., 2020).

In addition, the stereogenic center at position C5 of the oxazolidin-2-one ring is associated with its antibacterial activity (Li et al., 2014), and the oxidation of the morpholine ring is associated with the metabolic pathway of linezolid in the liver (Meng et al., 2015). The toxicity of linezolid, which leads to major adverse events in therapy, is largely due to its myelosuppression and MAO inhibition (Slatter et al., 2001). When the morpholine ring is replaced by other groups, such as the hydroxymethyl group, oxazolidinone antibacterial agents may have reduced effects on MAO and CYP450 subtypes (Zhao et al., 2021). In contrast to the morpholine heterocycle in linezolid, a 2,3- dihydropyridin-4-one (DHPO) ring has been introduced into the scaffold of contezolid, enhancing its antibacterial efficacy and significantly reducing its MAO inhibition (Nagiec et al., 2005). The main metabolic pathway of contezolid is not catalyzed by P450s but by multiple other enzymes, including flavin-containing dimethylaniline monooxygenase 5 (Meng et al., 2015). Consequently, the presence of a DHPO ring results in few clinically adverse drug–drug interactions between contezolid and P450 inhibitors or inducers.

## 3 Antibacterial efficacy

Given its antibacterial spectrum, it is likely that contezolid would cover most aerobic GPB. Similar to linezolid, contezolid could significantly reduce the bacterial load in the lungs in *Mycobacterium tuberculosis* Erdman-infected mice (Shoen et al., 2018). Contezolid showed almost the same antibacterial activity as linezolid in mouse models of systemic infection with strains of *Staphylococcus aureus, Streptococcus pneumoniae, E. faecalis,* and *Streptococcus pyogenes*, and mouse thigh infection model with *S. aureus* (Li et al., 2014).

In an *in vitro* antibacterial experiment against 1,211 GPB collected in the USA and Europe (Carvalhaes et al., 2020) and 1,321 clinical bacterial strains collected from 19 hospitals in China (Zhu et al., 2021), contezolid exhibited greater potency than linezolid: the antibacterial activity of contezolid against *Staphylococcus* was slightly better than that of linezolid, and it also showed the same effect against GPB as vancomycin and teicoplanin but had only a bacteriostatic effect on *Enterococcus* species. In addition, contezolid had good antibacterial activity against aerobic GPB, especially against the drug-resistant strains of such aerobic Gram-positive cocci as MRSA, penicillin-resistant *S. pneumoniae* (PRSP), and VRE. Of note, a previous study indicated that contezolid had limited activity against strains carrying linezolid-resistance genes, such as *Staphylococcus capitis* with the *cfr* gene and *E. faecalis* with the *optrA* gene, and *vanA*- and *vanM-*type VRE strains (Wang et al., 2021a). Moreover, compared with linezolid, contezolid was active against most non-tuberculous *Mycobacterium* reference strains, with the exception of *Mycobacterium avium* and *Mycobacterium intracellulare*, and pre-exposure to contezolid did not induce drug resistance (Wilcox, 2005; Guo et al., 2021).

## 4 Adverse reactions

Thrombocytopenia and anemia have been among the most important and common adverse effects associated with clinical treatment with linezolid since its first use in humans. To date, the mechanism responsible for linezolid-induced thrombocytopenia has not been clearly elucidated. The underlying pathogenic mechanisms of drug-induced thrombocytopenia include reduced platelet production due to bone marrow suppression and accelerated clearance of platelets from the circulation. However, it is not exclusively attributable to immune-mediated mechanisms (Danese et al., 2020).

Myelosuppression is directly associated with the mechanism of action of oxazolidinone antibiotics and is linked to the homology between the 23 S RNA target in prokaryotes and the genetically related mitochondrial protein synthesis machinery in mammals. As previously described, the novel structure of contezolid reduces its hematologic toxicity with long-term use. In a study of the incidence of drug-related hematologic abnormalities in patients treated with contezolid or linezolid, the safety of contezolid was evident (Hoy, 2021). After patients received more than 10 days of therapy, a significantly lower proportion of contezolid recipients (n = 204) than of linezolid recipients (n = 201) experienced a >30% reduction in platelet count, compared to baseline (2.5% vs. 25.4%; *p* < 0.001).

Furthermore, the neurotoxicity of oxazolidinone antibiotics manifests mainly as serotonin syndrome, particularly when co-administered with selective serotonin reuptake inhibitors, tricyclic antidepressants, or MAO inhibitors. In rodent models, contezolid demonstrated a 2- and 148-fold reduction in reversible inhibition of human MAO-A and MAO-B enzyme isoforms, respectively, compared to linezolid (Wang et al., 2021b). Moreover, no neurotoxicity and a significant increase in arterial blood pressure were observed for contezolid with oral doses of 120 mg/kg in both the mouse head-twitch model and the rat tyramine-challenge model.

## 5 Case reports

### 5.1 Case 1

A 57-year-old female patient with a 7-year history of systemic lupus erythematosus who had been taking a large number of oral hormonal and immunosuppressive drugs for a long time reported that a mushroom-like hard mass with a diameter of 6 cm had appeared in the upper part of her left leg 4 months earlier and that she had developed sudden chest pain, wheezing, and dyspnea 3 months ago. The patient was diagnosed with lower extremity cellulitis at the local hospital. A chest CT showed multiple nodules in both lungs, and a wraparound left pleural effusion. *Nocardia* infection foci were found in the affected parts of the lungs, intracranially, and in the lower limbs. Her symptoms of headache, nausea, vomiting, and intracranial hypertension were considered to be due to a craniocerebral *Nocardia* infection. Plain and contrast-enhanced brain magnetic resonance imaging showed more infections in the left frontal lobe.

On admission, her platelet count was normal (214 × 10^9^/L) before starting anti-infective treatment. She was started on linezolid. On day 7 of hospitalization, the patient’s white blood cell count reached 2.54 × 10^9^/L, and the erythrocyte count remained below the normal value (3.9 × 10^12^/L). Her platelet count reached 203 × 10^9^/L, which was still within the normal range but showed a decreasing trend as compared with the level at admission. Considering that this might reflect an adverse reaction to linezolid, the patient was given a human granulocyte-stimulating factor temporarily, and the treatment was changed to contezolid (800 mg po, 1/12 h) for 26 days. The decrease in platelet count continued until day 17 post-admission, when it reached 161 × 10^9^/L. With the use of contezolid, it gradually recovered from day 20 and returned to 202 × 10^9^/L on day 38. On day 37, after treatment with contezolid, the patient’s symptoms and various indicators were relatively stable, and her hematological indicators improved. Therefore, for pharmacoeconomic reasons, the patient began to use linezolid oral tablets. However, on day 50, the patient’s white blood cell count dropped to 2.67 × 10^9^/L, and her hemoglobin value continued to fluctuate around 100 g/L, while her platelet count decreased again to 100 × 10^9^/L, approaching the critical level. Therefore, on day 50, the patient resumed contezolid for 14 days until hospital discharge. Interestingly, on day 55, her platelet count rose again to 200 × 10^9^/L, approaching the pre-treatment level after admission.

It was necessary to maintain treatment for an adequate duration to ensure efficacy. A chest CT showed that the area of pulmonary inflammation was reduced. The platelet level decreased after the patient used linezolid but improved again after the patient used contezolid. Considering the time correlation, we believe that contezolid may have reduced the hematologic toxicity caused by linezolid, without affecting the anti-infective effect.

### 5.2 Case 2

A 65-year-old female patient was admitted to the hospital for abdominal distension and difficulty in defecation and was diagnosed with primary biliary and intestinal tumors, complicated by poor liver and kidney function. The patient was transferred to surgery for further robotic radical resection of hilar cholangiocarcinoma. Her wound recovery was poor after surgery, with successive severe mycosis, pulmonary infection, and ascites, with infections caused by *Enterococcus faecium*, *Klebsiella pneumoniae* and *Stenotrophomonas maltophilia*. Although the patient received albumin, thrombopoietin, and multiple blood transfusions, she remained anemic due to postoperative bleeding.

On day 17, the patient began taking linezolid for 12 days, and her platelet count decreased from 189 × 10^9^/L to 49 × 10^9^/L. However, the inflammatory indicators were significantly improved after the application of linezolid, and here the white blood cell count gradually decreased to its normal value, which indicated that linezolid was a reasonable treatment in this scheme. The pulmonary infection lesions on the bedside chest radiograph were reabsorbed, and the antibacterial treatment appeared to be effective. The patient then underwent multiple courses of continuous renal replacement therapy (CRRT) and blood transfusions. However, the patient’s inflammatory indicators were significantly improved after the use of linezolid, and the white blood cell count gradually decreased to the normal level, indicating that linezolid is reasonable in this scheme. Considering the patient’s serious illness, unstable hematology, multiple blood transfusions, etc., the decrease in platelets led to the withdrawal of linezolid on day 31 after admission, and she was started on contezolid tablets (800 mg, 1/12 h), until day 46. During this period, the patient improved after treatment and was transferred from the intensive care unit to the general ward. From day 33 to day 46, the patient’s inflammatory indicators remained relatively stable, the leukocyte indicators remained at normal values, the temperature was well-controlled, and her liver and kidney functions were stable. However, with the intractable mycosis, the patient suffered from a severe lung infection with shock and finally developed respiratory failure and cardiac insufficiency, and her condition became critical again. Considering the demands of the patient’s family, the patient chose to leave the hospital at that time and was lost to follow-up.

In this case, we did not observe that contezolid increased the burden on her liver and kidney functions, and inflammation-related indicators were well controlled, particularly once the white blood cell count was within the normal range. After the patient’s bleeding was controlled, her platelet status recovered significantly. Although other types of antibiotics were increased or decreased during this period, depending on the patient’s inflammatory indicators and drug-sensitivity test results, we still believe that contezolid was beneficial in controlling her inflammation. However, due to the worsening of respiratory failure, fungal pneumonia, and septic shock, her final condition was poor.

### 5.3 Case 3

An 86-year-old man was a tumor patient with a definite pulmonary MRSA infection. He had had a long history of sputum expulsion and wheezing, and his symptoms had gradually worsened in recent months. Upon admission, a pulmonary CT showed that there was a lesion in the lower lobe of the left lung, and he was subsequently diagnosed with lung cancer after an examination of tumor markers. The patient’s sputum bacterial culture showed a *S. aureus* infection. The patient was considered to be in the acute stage of infection, and anti-tumor treatment was not started. At that time, the patient’s platelet and white blood cell counts were within the normal range, but the red blood cell count was lower than the normal value. Therefore, based on the results of the drug-sensitivity test, tigecycline was added to his antibacterial treatment plan. However, on day 12 after admission, the patient’s chest radiograph showed no significant improvement in the lungs; for this reason, tigecycline was replaced with linezolid.

On day 16, the patient’s alveolar lavage fluid and sputum culture results indicated an MRSA infection. The patient’s antibacterial regimen was then changed to meperidine + levofloxacin + linezolid. After the addition of linezolid, his inflammatory indicators improved to some extent. However, the patient’s platelet count decreased from 286 × 10^9^/L to 201 × 10^9^/L. On day 20, the patient purchased contezolid tablets. The decreasing trend in the platelet count was corrected, from a low of 161 × 10^9^/L on day 24–365 × 10^9^/L on day 25. At the same time, infection-related indicators remained relatively stable, and C-reactive protein and white blood cell levels were in the normal range. On day 35, based on the chest CT findings, the patient’s improved pulmonary inflammatory status, and stable vital signs, the drug was stopped after 15 days of treatment.

In this case, after using linezolid, the MRSA infection was significantly improved, but the patient’s platelet level showed a downward trend, although it was not out of the normal range. For safety reasons, the doctors used contezolid, after which his platelet count stabilized. This patient illustrates that contezolid may have better safety in patients who require anti-infective treatment while having a tumor at the time of the acute infection period.

## 6 Discussion

In the three typical cases presented here, pulmonary inflammation, caused by infections with *Nocardia*, *Enterococcus faecium*, *Klebsiella pneumoniae*, *Stenotrophomonas maltophilia,* or MRSA, improved to varying degrees after the use of linezolid. However, linezolid induced thrombocytopenia in all patients after a few days and up to 1 week. Replacing this drug with contezolid improved the hematologic toxicity caused by linezolid, particularly the decrease in platelet levels. At the same time, it did not produce obvious impairment of liver and kidney function. Clinicians need a clear reason to adopt contezolid in terms of patient outcomes, patient safety, and health economics. In patients requiring anti-infective treatment, long-term use of oxazolidinone may be discontinued due to its hematologic toxicity, and close monitoring of hematologic indicators is needed. Although thrombocytopenia caused by linezolid is not an absolute outcome, once it occurs, it necessitates discontinuation of the treatment, even if it has a therapeutic effect. For example, patients with *Nocardia* infection must take oxazolidinone antibiotics for several months. Continuing treatment after discharge is more dangerous for some high-risk patients, such as older patients, those with tumors, or those at risk of bleeding, because their platelet levels cannot be monitored in time. A rational reason for replacing linezolid with contezolid may be to alleviate the blood toxicity of the former, such as the decrease in platelet levels.

Although some of the described patients’ anti-infective treatment plans included many other types of antibiotics, we did not find any time-related causality with their platelet decline. Interestingly, we did not observe an improvement in thrombocytopenia in patients taking contezolid in other circumstances, such as patients with vancomycin-induced thrombocytopenia. Although the mechanism underlying contezolid’s correction of thrombocytopenia is still unknown, the hypothesis is supported by the observation that platelet levels were significantly improved, which sets its effects apart from those of other antibiotics. In general terms, we believe that it is reasonable to expect that contezolid can improve the platelet decrease caused by linezolid.

Almost one-third of patients treated with linezolid for more than 10 days or more than 14 days develop thrombocytopenia as an adverse reaction. The likelihood of its occurrence increases with the duration of linezolid use (Attassi et al., 2002). This leads not only to temporary adjustments in the treatment plan but will also lead to potential adverse consequences for those patients who continue to take anti-infective drugs after discharge. To date, the mechanism by which linezolid induces thrombocytopenia has not yet been elucidated. In the application of oxazolidinone antibiotics, adverse reactions remain a major restriction that needs to be considered. Although there are currently no randomized trials to evaluate whether contezolid can be used as a substitute for linezolid, we hope that the clinical application of contezolid, particularly its safety, will lead to consideration of contezolid as a replacement therapy for linezolid, particularly in patients who require long-term use to control and improve pulmonary infection. We believe that, given the need for new oxazolidinone antibiotics with reduced myelosuppression or development of thrombocytopenia, contezolid has good prospects for clinical application. Further work on the safety and efficacy of the clinical use of contezolid would be beneficial.

## Data Availability

The original contributions presented in the study are included in the article/Supplementary Material, further inquiries can be directed to the corresponding author.
